# The Impact of Preoperative Dexamethasone Administration on Quality of Recovery Following Laparoscopic Sleeve Gastrectomy: A Prospective Observational Study

**DOI:** 10.1007/s11695-024-07121-8

**Published:** 2024-03-12

**Authors:** Mehmet Gokhan Taflan, Yasemin Burcu Ustun, Esra Turunc, Cengiz Kaya, Burhan Dost, Sezgin Bilgin, Emine Ozdemir, Gokhan Selcuk Ozbalci

**Affiliations:** 1 Department of Anesthesiology and Reanimation, Faculty of Medicine, Ondokuz Mayis University, Samsun, TR55139 Turkey; 2 Department of General Surgery, Faculty of Medicine, Ondokuz Mayis University, Samsun, Turkey

**Keywords:** Obesity, Laparoscopic sleeve gastrectomy, Dexamethasone, Postoperative recovery quality, QoR40

## Abstract

**Introduction:**

Recovery from anesthesia is complex and affected by multiple factors. In patient with obesity, the increased prevalence of anxiety and depressive disorders poses a challenge in achieving optimal patient satisfaction. Therefore, strategies to enhance the quality of recovery are crucial for this population. This study aimed to investigate whether administration of dexamethasone to patients undergoing laparoscopic sleeve gastrectomy (LSG) could improve recovery outcomes.

**Methods:**

This prospective observational study was conducted at a tertiary university hospital in Samsun, Turkey. Thirty patients who received dexamethasone prior to LSG (group D) and 30 patients who did not (group C) were included with convenience sampling method. The quality of recovery was assessed using the Quality of Recovery 40 questionnaire (QoR-40). The primary outcome measure was the QoR-40 score at 24 h postoperatively.

**Results:**

The dexamethasone group showed a significant improvement in QoR-40 scores (185.4 ± 6.0 vs. 172.0 ± 8.4, *p* < 0.001), exhibited reduced morphine consumption (11.8 ± 7.8 vs. 21.8 ± 10.9 mg, *p* < 0.001), opioid demand count (21.50 [9.50–49.00], *p* = 0.001), the number of patient used antiemetic drug (1 vs. 22, *p* < 0.001), and achieved earlier mobilization (3 [3–4] vs. 3 [3–4] h, *p* < 0.0001). However, no significant differences were observed between the two groups concerning intraoperative complications, postoperative wound infections, or time to discharge.

**Conclusions:**

In patients undergoing laparoscopic sleeve gastrectomy, preoperative dexamethasone administration was associated with improved the recovery quality after discharge and reduced early postoperative need for antiemetic medications.

**Graphical Abstract:**

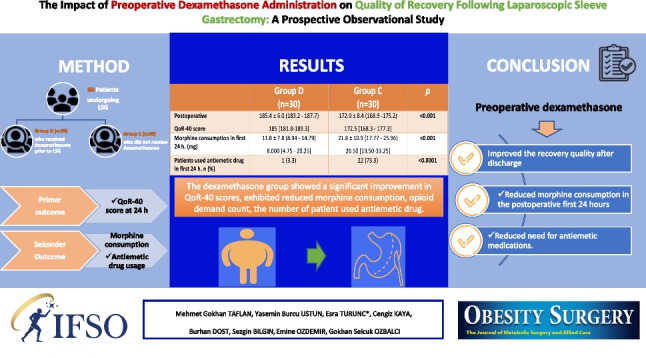

## Introduction

Obesity is a significant public health concern with rising prevalence worldwide that has led to an increasing number of bariatric surgeries being performed as a treatment option. Postoperative recovery in patients with obesity is of paramount importance; however, achieving patient satisfaction in this specific population can be challenging. The morbidity and mortality associated with surgery have decreased compared to that in the past, and there is an increasing focus on patient-centered outcomes, such as recovery quality. However, numerous factors present a challenge, including opioid-induced respiratory depression, a higher incidence of postoperative nausea and vomiting (PONV) [1], and the comorbidities such as anxiety and depression [2, 3].

The Quality of Recovery-40 (QoR-40) questionnaire is a comprehensive assessment tool that evaluates postoperative recovery across various dimensions, including physical, emotional, cognitive, and functional improvements following surgery. This facilitates the development of tailored solutions for individual patients, thus enhancing perioperative care management.

Dexamethasone, a synthetic steroid that has long been used for its anti-inflammatory effects is widely approved as a standard prophylactic and therapeutic measure for PONV [4]. Moreover, dexamethasone promotes postoperative analgesia [5–7].

This study aimed to investigate the effects of dexamethasone on postoperative recovery following laparoscopic sleeve gastrectomy (LSG) in patients with obesity. We hypothesized that there would be a significant difference in 24-h QoR-40 scores between patients who received dexamethasone and those who did not. Additionally, we aimed to determine the incidence of PONV, opioid consumption, and need for antiemetics in patients receiving dexamethasone.

## Materials and Methods

### Study Design

This prospective, single-center, observational, cohort, two-parallel-arm study was conducted in accordance with the principles outlined in the Declaration of Helsinki [8] after obtaining approval from the Ondokuz Mayis University Clinical Research.

Ethics Committee (approval no: 2021/560) and the Ministry of Health of Turkey (22-AKD-181–03.07.2020). The study was registered at ClinicalTrials.gov (NCT05752734) before the enrollment of any participant, and written informed consent was obtained from all participants for the registration and publication of data. In the study, 30 patients who received dexamethasone prior to LSG (group D) and 30 patients who did not (group C) were included using a convenience non-probability sampling method. While both the researcher and outcome assessors were blinded, the patients were not blinded in our study.

Patients who met the following criteria were enrolled between February 2023 and June 2023: scheduled for LSG, body mass index (BMI) > 30 kg/m^2^, aged 18–65 years, and an American Society of Anesthesiologists (ASA) classification of II–III. The Strengthening the Reporting of Observational Studies in Epidemiology (STROBE) checklist was followed [9].

Patients with diabetes mellitus, renal, cardiac, and liver diseases, psychiatric and neurological conditions, a history of alcohol and substance abuse, patients who have been using non-invasive positive airway pressure therapy at home (apnea–hypopnea index (AHI) > 5/h with symptoms as diagnosed OSA with polysomnography), and those who did not provide consent were excluded from the study.

### General Anesthesia

During the preoperative visit, each patient was informed regarding the use of the patient-controlled analgesia (PCA) device and QoR-40 questionnaire. Patients were also briefed on the use of numerical rating scale (NRS) for pain severity (0, no pain; and 10, most severe pain).

A standard protocol created for this patient group was applied for management of anesthesia in all patients who underwent LSG in our hospital. Accordingly, certain patients received 8 mg IV dexamethasone 1 h before the surgery in the preoperative waiting area, based on the anesthesiologist’s choice. Patients who fasted overnight were placed in the ramp position on the operating table, and electrocardiography (EKG), oxygen saturation (SpO2), and non-invasive blood pressure monitoring were performed in accordance with ASA standards.

After monitoring, patients were first preoxygenized and then intubated by administering iv remifentanil infusion (0.1–0.25 mcg/kg/min), propofol (1.5–2 mg/kg), and rocuronium bromide (0.6 mg/kg) for muscle relaxation. The drugs were administered according to body weight, calculated using the formula [ABW: ideal body weight (IBW) + 0.4 × (actual body weight-IBW)].

Anesthesia was maintained with an oxygen–air mixture (FiO2 0.50), sevoflurane, and remifentanil infusion 0.1–0.25 mcg/kg/min. Mechanical ventilator settings were tidal volume 6–8 ml/kg IBW, inspiratory/expiratory ratio 1:2, and respiratory rate EtCO2 30–38 mmHg. The remifentanil infusion rate was set to allow up to a 20% change in the patients’ preinduction heart rate and mean blood pressure. After induction, tramadol 100 mg and tenoxicam 20 mg iv were administered, and iv morphine 0.05 mg/kg/IBW was added during the intraoperative period. Thirty minutes before the end of the surgical procedure, an intravenous infusion of paracetamol (1 g) and ondansetron (8 mg) was administered. At the end of the surgery, neostigmine (0.03 mg/kg) and atropine (0.015 mg/kg) were administered to reverse the effects of rocuronium, and the patient was extubated. When there was a change of > 20% in the hemodynamic parameters of the patients during the intraoperative period, sympathomimetic/sympatholytic drugs such as esmolol, nitroglycerin, noradrenaline, and adrenaline were administered.

### Postoperative Period

PONV was evaluated using a verbal descriptive scale (0, none; 1, mild nausea; 2, moderate nausea; 3, vomiting once; 4, multiple vomiting). In the case of verbal scale > 2, metoclopramide 0.15 mg/kg was used as a rescue antiemetic. Patients with a modified Aldrete score of > 9 in the recovery unit were sent to the general surgery ward with a morphine PCA device with the following settings: bolus dose, 1 mg; lock-out time, 15 min, 4-h limit, 80% of the calculated total dose. During the follow-up of these patients, paracetamol 1 g was administered thrice a day at 8-h intervals. Patients were informed that they could request opioids through the PCA device if their NRS score was > 3. Patients with an NRS score > 4 despite opioid administration with the PCA device were administered tramadol 0.5 mg/kg/IBW. The 24-h morphine consumption was defined as the net amount of morphine consumed through PCA. This definition excluded any other opioids used perioperatively. The mobilization time was defined as the period between admission to the ward and when the patient could sit on a chair without assistance. Hospital discharge instructions were used to determine whether patients were ready for discharge.

### Clinical Endpoints and Variables

Patient characteristics, demographic information, and other data were obtained from the electronic medical records and patient files. A resident who was not participated in the study performed all the assessment. The primary objective of our study was to measure the QoR-40 score 24 h postoperatively. The QoR-40 questionnaire consists of a total of 40 questions in 5 subdomains: physical independence (*n* = 5), patient support (*n* = 7), comfort (*n* = 12), emotional status (*n* = 9), and pain (*n* = 7). Each question is rated on a Likert scale ranging from 1 to 5, and the sum of these scores yields a total score ranging from 40 (worst recovery quality score) to 200 (best recovery quality score). Two groups were formed on the basis of the use of steroids for multimodal analgesia. Sampling was performed using a convenience non-probability sampling technique, taking into consideration the inclusion criteria. According to this sampling method, patients need to be willing to participate in the study initially. The assignment of consenting patients to the experimental group is entirely determined by the preference of the anesthesiologist in charge of anesthesia management.

All patients were administered the QoR-40 questionnaire at 24 h postoperatively. The secondary objectives of our study were to assess the number of opioid demand count via PCA device and consumption of morphine first 24 h postoperatively, the incidence of patient who need rescue analgesics and antiemetics, intraoperative complications, postoperative wound infection, time to mobilization, and time to discharge.

All patients were provided with analgesia control through a PCA device for 24 h. At the end of 24 h, patients were evaluated using the 40-item QoR-40 questionnaire. A resident who was not participated in the study performed all the assessment.

The sample size for inclusion was determined using power analysis. G Power 3.1.9.2 software was used for the *t*-test of independent samples, considering a previous study’s global QoR-40 scores (group treatment: 182.1 ± 12 vs. group control: 183.7 ± 9). With a significance level of 5%(α), test power of 80% (1-β), effect size of *d* = 0.790, and a two-tailed hypothesis, the minimum sample size required for each group was determined to be 27, resulting in a total of 54 participants [10]. Considering potential data loss, 60 patients were included in the study.

### Statistical Analysis

Descriptive statistics, frequencies, and other features were used for statistical analysis of the patient data, including all variables. Continuous data are presented as mean ± standard deviation (95% confidence interval (CI)) or median (Q1–Q3). Continuous variables were analyzed using Shapiro–Wilk and Kolmogorov–Smirnov tests to determine the normal distribution of data. Continuous and normally distributed variables were compared using Student’s *t*-test. Nonparametric tests were used when the data did not follow a normal distribution. Categorical variables were assessed using the chi-square test, and in some cases, Fisher’s exact test was applied. Logistic regression tests were used to analyze risk factors. Analyses were conducted using SPSS Statistics for Windows (version 21.0; IBM Corp., Armonk, NY, USA). All *p*-values were two-tailed, and *p* ≤ 0.05 was considered statistically significant. Non-normally distributed quantitative data included subscores for emotions, physical independence, patient support, pain, BMI, morphine consumption in the first 24 h, and morphine demand count.

## Results

**Fig. 1:**
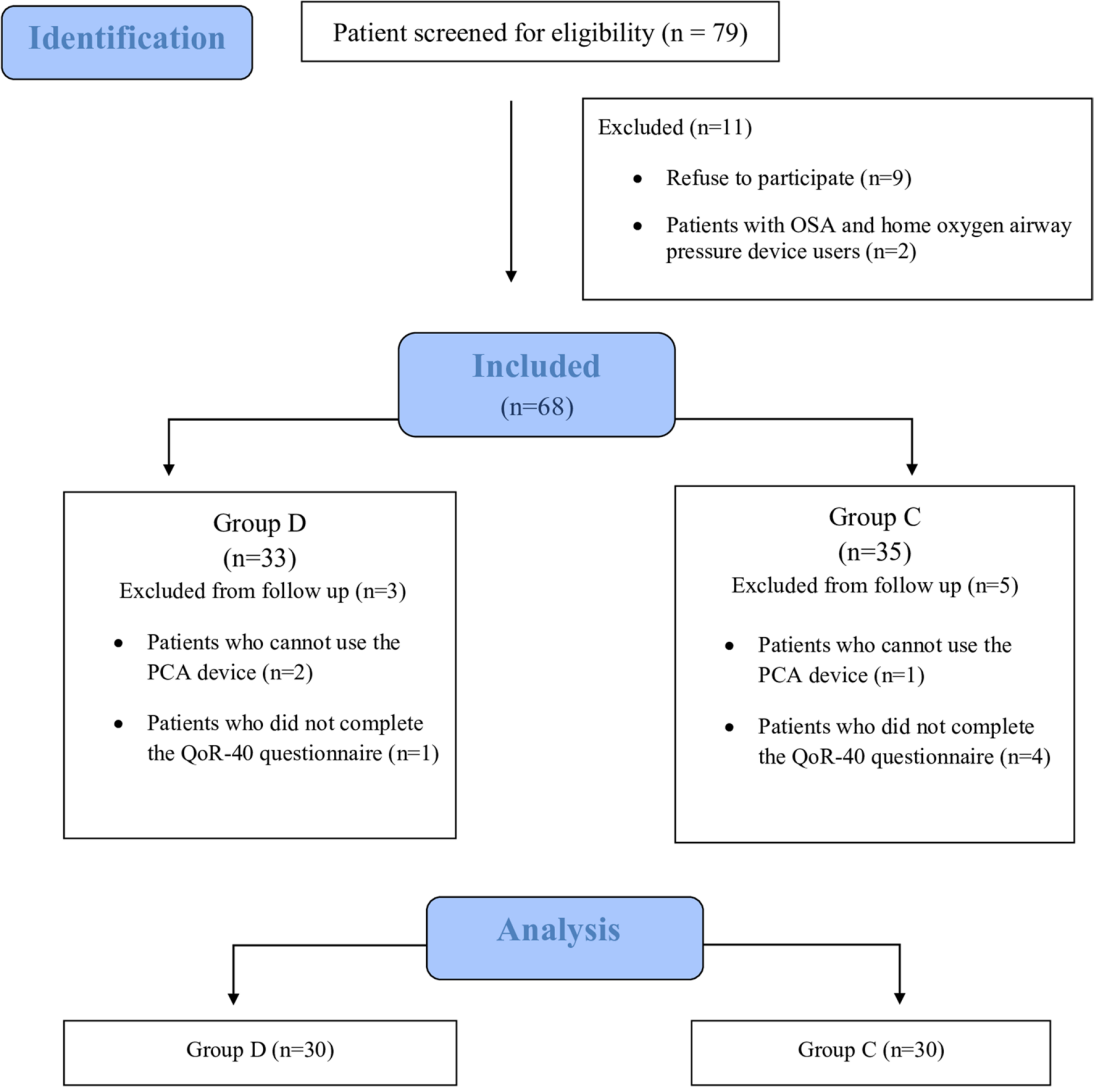
Flow diagram showing the distribution of patient data. Abbreviations: OSA, obstructive sleep apnea; PCA, patient controlled analgesia; QoR-40, Quality of Recovery-40

In this study, 79 patients initially screened. Of the enrolled patients, 11 were excluded for the following reasons: nine patients decided to leave the study, two patients with obstructive sleep apnea and home oxygen device users. Sixty-eight patients who met the inclusion criteria were included in the study. During the follow-up, three patients in the dexamethasone group and five patients in the control were excluded. Ultimately, data for 60 patients were analyzed (Fig. 1).

There were no significant differences in demographic data between the groups (Table 1). The QoR-40 score was higher in the group D (*p* < 0.001). Considering the subdomains of the QoR-40 score, group D exhibited higher scores for comfort, emotions, physical independence, patient support, and pain (Table 2).

**Table 1:** Patient demographic and surgical characteristics and clinical outcomes

	Group D (*n* = 30)	Group C (*n* = 30)	*p*
Sex, female/male *n (%)*	26 (86)/4 (13.3)	25 (83)/5 (16.6)	0.718
ASA (II/ III), *n (%)*	20 (66.6)/10 (33.3)	19 (63.3)/11 (36.6)	1.00
	mean ± SD (95% CI) median [Q1–Q3]	mean ± SD (95% CI) median [Q1–Q3]	
Age, years	31.9 ± 8.1 (28.86–34.94) 31.00 [27.00–35.00]	35.7 ± 12.1 (31.21–40.26) 33.00 [26.75–45.50]	0.157
BMI, kg/m^2^	46.8 ± 17.3 (40.25–48.80) 42.50 [38.00–45.80]	42.7 ± 6.6 (40.32–45.26) 41.05 [37.45–45.90]	0.236
Duration of anesthesia (min)	84.5 ± 9.4 (80.96–87.97) 85.00 [80.00–90.00]	85.6 ± 14.5 (80.15–90.98) 86.50[ 75.00–90.00]	0.729
Duration of surgery (min)	74.7 ± 8.3 (71.60–77.80) 73.50 [70.00–77.75]	75.7 ± 13.7 (70.54–80.79) 75.00 [65.00–80.00]	0.742
Time to first mobilization (h)	3.2 ± 0.85 (2.89–3.50) 3.00 [3.00–4.00]	3.9 ± 0.6 (3.67–4.12) 3.00 [3.00–4.00]	**< 0.0001**
Time to discharge (day)	2.97 ± 0.18 (2.89–3.03) 3.00 [3.00–3.00]	3.03 ± 0.18 (2.96–3.03) 3.00 [3.00–3.00]	0.231

Continuous variables are presented as median [Q1–Q3] or mean ± standard deviation (95% CI) and categorical variables are presented as counts (%). Statistically significant difference is highlighted in bold

*ASA* American Society of Anesthesiologists, *BMI* body mass index, *CI* confidence interval, *SD* standard deviation

**Table 2:** QoR-40 outcomes at 24 h postoperatively

	Group D (*n* = 30)	Group C (*n* = 30)	*p*
	Mean ± SD (95% CI) Median [Q1–Q3]	Mean ± SD (95% CI) Median [Q1–Q3]	
Postoperative QoR-40 score	185.4 ± 6.0 (183.2–187.7) 185 [181.8–189.3]	172.0 ± 8.4 (168.9–175.2) 172.5 [168.3–177.3]	**< 0.001**
Comfort	56.47 ± 2.01 (55.72–57.22) 56.50 [54.75–58.00]	51.97 ± 3.50 (50.66–53.28) 52 [50.00–54.00]	** < 0.001**
Emotional state	42.27 ± 1.68 (41.64–42.89) 42 [42.00–43.00]	39.53 ± 2.54 (38.58–40.48) 40 [38.75–42.00]	0.035
Physical independence	20.50 ± 2.19 (19.68–21.32) 20 [19.00–21.00]	18.53 ± 3.02 (17.40–19.66) 17.50 [16.75–22.00]	**0.006**
Patient support	31.53 ± 3.13 (30.36–32.70) 33.00 [29.00–34.25]	29.43 ± 2.56 (28.47–30.39) 29.00 [28.00–29.00]	**0.012**
Pain	34.57 ± 1.00 (34.19–34.94) 35.00 [35.00–35.00]	32.57 ± 1.59 (31.97–33.16) 33.00 [32.00–34.00]	** < 0.001**

Data are presented as median [Q1–Q3] and mean ± standard deviation (95% CI). Statistically significant difference is highlighted in bold

*CI* confidence interval, *SD* standard deviation

In the postoperative period, group D had an opioid demand count of (median [Q1–Q3]), 21.50 [9.50–49.00], and an average morphine consumption of (median [Q1–Q3]), 8 [4.750–20.25] mg, whereas group C had an opioid demand count of (median [Q1–Q3]), 79 [22.75–125.0], and an average morphine consumption of (median [Q1–Q3]), 20.50 [13.50–33.25] mg (*p* = 0.001 and *p* < 0.001, respectively). The requirement for antiemetic medication and rescue analgesics were significantly lower in group D than in group C (*p* < 0.0001 and *p* = 0.015, respectively). There was no difference between the groups regarding remifentanil consumption (Table 3).

**Table 3:** Comparison of opioid and antiemetic consumption in the first 24 h postoperatively among study groups

	Group D (*n* = 30)	Group C (*n* = 30)	*p*
	Mean ± SD (95% CI) median [Q1–Q3]	Mean ± SD (95% CI) median [Q1–Q3]	
Opioid demand count	32.1 ± 28.1 (21.56–42.57) 21.50 [9.50–49.00]	87.97 ± 69.70 (61.94–114.0) 79.00 [22.75–125.0]	**0.001**
Morphine consumption in first 24 h. (mg)	11.8 ± 7.8 (8.94–14.79) 8.000 [4.75–20.25]	21.8 ± 10.9 (17.77–25.96) 20.50 [13.50–33.25]	** < 0.001**
Patients used antiemetic drug in first 24 h. n (%)	1 (3.3)	22 (73.3)	** < 0.0001**
Intraoperative remifentanil consumption (μg)	688 ± 261 (590.7–785.3) 600 [515.0–810.0]	643 ± 288 (535.0–750.3) 620 [430.0–785.0]	**0.574**
Patients given rescue analgesic in first 24 h, *n (%)*	22 (73.3)	29 (96.6)	**0.015**

Continuous variables are presented as median [Q1–Q3] or mean ± standard deviation (95% CI) and categorical variables are presented as counts (percentages) (%). Statistically significant difference is highlighted in bold

*CI* confidence interval, *SD* standard deviation

No intraoperative complications or postoperative wound infections were observed in any of the patients. There was no significant difference between the groups regarding time to discharge. However, the group D demonstrated significantly earlier postoperative mobilization (*p* < 0.0001) (Table 1).

## Discussion

Our study showed that pre-operative 1-h dexamethasone administration was associated with improved recovery quality, reduced opioid, and antiemetic requirements.

We obtained a higher global QoR-40 questionnaire score with dexamethasone in the scale consisting of five subheadings. Dexamethasone improves the recovery scores in laparoscopic cholecystectomy [11], cardiac [12], and vaginal surgery [13]. However, another study reported different results in lower-extremity surgery with spinal anesthesia [14]. This may be due to the impairment of patient comfort caused by intrathecal morphine-related postoperative itching, PONV. The results of another study, in which only a local anesthetic was used for spinal anesthesia, were similar to ours [15].

The subheadings of the QoR-40 questionnaire include emotions, comfort, physical independence, patient support, and pain. The relationship between steroid administration and mood changes has been investigated previously [16, 17]. Manic symptoms are more common after acute steroid treatment and depressive symptoms are more common after long-term steroid use [18–20]. The emotional improvement in the dexamethasone group may have been due to the neuropsychological effects of steroids. Considering the emotional components of obesity and eating disorders, improvement in the emotions subheading from the patient’s perspective is valuable. The QoR-40 scale evaluates “nausea, vomiting, able to communicate with hospital staff, family, or friends, has normal speech, and able to write” in the comfort, physical independence, and patient support subheadings. Improvement in pain symptoms by dexamethasone can explain the positive differences between these subheadings. The decrease in opioid use can increase patient communication with the environment, physical independence, and patient support scores.

Pain is another assessment of the QoR-40 questionnaire. Dexamethasone has been studied in multimodal analgesia models and is included in pain management guidelines developed for different surgeries [5–7]. Participants in the dexamethasone group had lower pain scores, which is consistent with the results of previous studies [21, 22]. Therefore, we observed lower postoperative morphine consumption, because dexamethasone reduces acute inflammation induced by tissue damage.

LSG is associated with PONV, which not only affects patient comfort, but can also lead to delayed discharge from the post-anesthesia care unit [23]. Dexamethasone has been approved as a standard prophylactic and therapeutic agent for PONV [4]. Consistent with literature, our study showed that patients in the steroid-treated group had a reduced need for antiemetics. The decrease in opioid consumption may have contributed to these outcomes. The discrepancy in results between our study and those of Bataille et al., who found equivalent efficacy between dexamethasone and ondansetron using a placebo, could be attributed to the lower dose of dexamethasone (4 mg) they used. Additionally, the use of total intravenous anesthesia instead of inhalation agents in our study may have influenced the incidence of PONV, considering propofol’s antiemetic properties [24]. Glucocorticoids primarily exert their effects by binding to intracellular receptors and causing changes in gene transcription [25]. Therefore, the biological effects usually start within 1–2 h. We administered dexamethasone 1-h before surgical trauma, which may explain the improved results in terms of analgesic effect and prevention of PONV, compared to administering it immediately before induction. The opioid-reducing and antiemetic effects provided by dexamethasone can be further enhanced by complementing them with opioid-sparing anesthetic techniques. This way, particularly in comorbid patients, avoiding opioid side effects can lead to a greater increase in patient satisfaction and facilitate early discharge.

There were no intraoperative complications or postoperative wound infections related to dexamethasone use. Although the postoperative mobilization time significantly decreased in the dexamethasone group, due to the improvement in the quality of postoperative recovery, the time to discharge was similar between the two groups. Reports have indicated that dexamethasone shortens hospital stay [12]; however, further studies with adequate sample sizes and power analyses are required to confirm this finding.

Our study had some limitations. First, we did not administer the QoR-40 questionnaire to patients in the preoperative period, preventing a direct comparison between preoperative and postoperative values. Second, we administered the scale only once during the postoperative period; thus, the duration of the positive effects of dexamethasone is unclear. Third, we administered the standard dose of dexamethasone, which is considered safe and within our clinic’s protocol, without individualizing it based on the patients’ obesity status. Fourth, we did not assess intraoperative and postoperative blood glucose levels, which could have been beneficial for evaluating insulin resistance and altered glycemic responses commonly observed in patients with obesity during the perioperative period. Fifth, postoperative wound infections were monitored only until the point of discharge. This may restrict our ability to capture and observe most occurrences of infections, as many of these complications tend to manifest post-discharge. The last one, we could not blind the patients due to nature of observational study design.

In conclusion, we observed that preoperative administration of 8 mg dexamethasone was associated with significantly improved quality of postoperative recovery and reduced the incidence of PONV in patients undergoing LSG. Nevertheless, randomized controlled trials evaluating different doses of dexamethasone in patient with obesity are necessary to assess their impact on the quality of recovery, side effect profile, and hospital stay.

## References

[1] Halliday TA, Sundqvist J, Hultin M (2017). Post-operative nausea and vomiting in bariatric surgery patients: an observational study. Acta Anaesthesiol Scand.

[2] Kalarchian MA, Marcus MD, Levine MD (2007). Psychiatric disorders among bariatric surgery candidates: relationship to obesity and functional health status. Am J Psychiatry.

[3] de Zwaan M, Enderle J, Wagner S (2007). Anxiety and depression in bariatric surgery patients: a prospective, follow-up study using structured clinical interviews. J Affect Disord.

[4] Gan TJ, Belani KG, Bergese S (2020). Fourth consensus guidelines for the management of postoperative nausea and vomiting. Anesth Analg.

[5] Roofthooft  E, Joshi GP, Rawal  N (2021). PROSPECT guideline for elective caesarean section: updated systematic review and procedure-specific postoperative pain management recommendations. Anaesthesia.

[6] Jacobs A, Lemoine A, Joshi GP (2020). PROSPECT guideline for oncological breast surgery: a systematic review and procedure-specific postoperative pain management recommendations. Anaesthesia.

[7] Barazanchi AWH, MacFater WS, Rahiri J-L (2018). PROSPECT collaboration. Evidence-based management of pain after laparoscopic cholecystectomy: a PROSPECT review update. Br J Anaesth.

[8] World Medical Association (2013). World Medical Association Declaration of Helsinki: ethical principles for medical research involving human subjects. JAMA.

[9] Ghaferi AA, Schwartz TA, Pawlik TM (2021). STROBE reporting guidelines for observational studies. JAMA Surg.

[10] Martins MJ, Martins CPMO, Castro-Alves LJ (2018). Pregabalin to improve postoperative recovery in bariatric surgery: a parallel, randomized, double-blinded, placebo-controlled study. J Pain Res.

[11] Murphy GS, Szokol JW, Greenberg SB (2011). Preoperative dexamethasone enhances quality of recovery after laparoscopic cholecystectomy: effect on in-hospital and postdischarge recovery outcomes. Anesthesiol.

[12] Murphy GS, Sherwani SS, Szokol JW (2011). Small-dose dexamethasone improves quality of recovery scores after elective cardiac surgery: a randomized, double-blind, placebo-controlled study. J Cardiothorac Vasc Anesth.

[13] Pauls RN, Crisp CC, Oakley SH (2015). Effects of dexamethasone on quality of recovery following vaginal surgery: a randomized trial. Am J Obstet Gynecol.

[14] Moro ET, Ferreira MAT, Gonçalves RDS, et al. The quality of recovery after dexamethasone, ondansetron, or placebo administration in patients undergoing lower limbs orthopedic surgery under spinal anesthesia using intrathecal morphine. A randomized controlled trial. Anesthesiol Res Pract. 2020;9265698. 10.1155/2020/9265698 PMC7256731 32518560

[15] Patnaik S, Singh S, Vivekanand D (2021). Evaluation of quality of recovery score in mothers and neonatal outcome assessment after surgery using preoperative dexamethasone for caesarean section. Med J Armed Forces India.

[16] Wolkowitz OM, Reus VI, Weingartner H (1990). Cognitive effects of corticosteroids. Am J Psychiatry.

[17] Wolkowitz OM, Rubinow D, Doran AR (1990). Prednisone effects on neurochemistry and behavior preliminary findings. Arch Gen Psychiatry.

[18] Naber D, Sand P, Heigl B (1996). Psychopathological and neuropsychological effects of 8-days’ corticosteroid treatment A prospective study. Psychoneuroendocrinology.

[19] Brown ES, Suppes T, Khan D (2002). Mood changes during prednisone bursts in outpatients with asthma. J Clin Psychopharmacol.

[20] Brown ES (2009). Effects of glucocorticoids on mood, memory, and the hippocampus. Treatment and preventive therapy. Ann N Y Acad Sci.

[21] Mitchell C, Cheuk SJ, O’Donnell CM (2022). What is the impact of dexamethasone on postoperative pain in adults undergoing general anaesthesia for elective abdominal surgery: a systematic review and meta-analysis. Perioper Med (Lond).

[22] Zhuo Y, Yu R, Wu C, et al. The role of perioperative intravenous low-dose dexamethasone in rapid recovery after total knee arthroplasty: a meta-analysis. J Int Med Res. 2021;49(3) 10.1177/0300060521998220 PMC7952853 33685282

[23] Ashoor TM, Kassim DY, Esmat IM (2022). A randomized controlled trial for prevention of postoperative nausea and vomiting after laparoscopic sleeve gastrectomy: aprepitant/dexamethasone vs. mirtazapine/dexamethasone. Anesthesiol Res Pract.

[24] Bataille A, Letourneulx JF, Charmeau A (2016). Impact of a prophylactic combination of dexamethasone-ondansetron on postoperative nausea and vomiting in obese adult patients undergoing laparoscopic sleeve gastrectomy during closed-loop propofol-remifentanil anaesthesia: a randomised double-blind placebo-controlled study. Eur J Anaesthesiol.

[25] Sapolsky RM, Romero LM, Munck AU (2000). How do glucocorticoids influence stress responses? Integrating permissive, suppressive, stimulatory, and preparative actions. Endocr Rev.

